# Comparability of semiautomatic tortuosity measurements in the carotid artery

**DOI:** 10.1007/s00234-018-2112-3

**Published:** 2018-10-18

**Authors:** Evelien E. de Vries, Vanessa E. C. Pourier, Constance J. H. C. M. van Laarhoven, Evert J. Vonken, Joost A. van Herwaarden, Gert J. de Borst

**Affiliations:** 1 Department of Vascular Surgery, University Medical Center Utrecht, Room G04.129, PO Box 85500, 3508 GA Utrecht, the Netherlands; 2 Department of Radiology, University Medical Center Utrecht, Utrecht, the Netherlands

**Keywords:** Carotid artery disease, Carotid aneurysm, Software validation, CT angiography, Quantitative analysis

## Abstract

**Purpose:**

Increased arterial tortuosity has been suggested as a predisposing factor for carotid artery dissection, which is an important risk factor for development of extracranial carotid artery aneurysms (ECAA). Prior to comparison with non-ECAA controls, the optimal measurement technique should be defined. This study describes the difference between software packages in terms of reproducibility and absolute outcome of arterial tortuosity measurements in ECAA patients.

**Methods:**

CT-angiography analysis was performed on 12 ECAA patients selected from our registry, using four software packages: 3mensio Vascular, TeraRecon, Vital Images, and Aycan OsiriX PRO. The tortuosity index (TI) was calculated from the skull base until the carotid bifurcation and aortic arch, and was defined as the centerline’s true length divided by the straight line distance. Intraclass correlation coefficients (ICC) with 95% confidence intervals were calculated to quantify inter- and intra-observer variability within one software package, and differences in measured TI between packages.

**Results:**

Inter-observer agreement was nearly perfect for 3mensio, excellent for Vital Images and OsiriX, and substantial for TeraRecon, with ICC 0.99 (0.96–1.0), 0.90 (0.69–0.97), 0.84 (0.53–0.95), and 0.72 (0.28–0.91), respectively. Intra-observer agreement ranged from ICC 1.0 for 3mensio to 0.91 for TeraRecon. Agreements in TI ranged from ICC 0.99 (0.98–1.0) for 3mensio vs. OsiriX, to 0.95 (0.82–0.98) for 3mensio vs. TeraRecon. Median time needed to complete one round of measurements was highest for OsiriX (*p* = 0.013).

**Conclusions:**

Carotid artery tortuosity measurements are reproducible and comparable between current commercially available software packages, with high intra-observer agreement. Although the reproducibility differed per software packages, all packages scored an acceptable inter-observer agreement.

## Introduction

Increased arterial tortuosity has been suggested as a predisposing factor for carotid artery dissection [1–3], which is in turn an important etiological risk factor for extracranial carotid artery aneurysm (ECAA) formation [4]. Conversely, although experts in the field have suggested that most ECAA have a relative elongated inflow and outflow track, no validated data exist about arterial tortuosity in ECAA patients. In fact, little is known about the natural clinical course and risk factors for adverse outcome of ECAA patients [5]. If increased arterial tortuosity would exist in ECAA patients, it may aid in individual patient’s risk prediction for adverse outcome. In addition, severe tortuosity affects planning and performing interventions for aneurysm exclusion when indicated [6].

In order to validate the relative tortuosity in patients with ECAA as compared to patients with normal carotid arteries, a comparative study with non-ECAA controls should be set-up. Beforehand, it is crucial to first establish a standardized method to define and measure the arterial tortuosity. Tortuosity is defined in literature as the property of the artery to have “many turns” [7]. Multiple definitions exist to quantify arterial tortuosity, but due to its good reproducibility values, it is commonly referred to as the tortuosity index (TI), which is the true length of the vessel divided by the straight distance [8–11].

Manual measurements in different arterial territories are reliable, but time consuming [12–14]. Multiple software packages to facilitate (semiautomatic) TI measurements are commercially available. It is unknown how the tortuosity measurements performed by these different packages relate to each other in terms of inter- and intra-observer variability, and differences in absolute measured tortuosity. Furthermore, it is unknown how increased vascular tortuosity will influence the assessed values within these software packages.

Accordingly, the present study aimed to investigate the difference between these software packages in terms of reproducibility and absolute outcome of carotid artery tortuosity measurements, in patients with an ECAA.

## Methods

### Case selection

Datasets of 12 patients with an ECAA all located in the internal carotid artery (ICA) were retrieved from our Carotid Aneurysm Registry (www.carotidaneurysmregistry.com) [15]. The registry has been approved by the local ethics committee, and all patients gave informed consent. For the purpose of this study, a computed tomography angiography (CTA) scan with slice thickness below 1.0 mm was eligible for inclusion to guarantee proper slice thickness for reconstruction. This necessary condition limited the amount of eligible CTAs to 12 due to rarity of disease. The CTAs had been performed for evaluation or treatment of ECAA between 2008 and 2017, in the University Medical Center Utrecht. We aimed to select an equal amount of cases with fusiform and saccular ECAA. As specified within the registry protocol [15], fusiform aneurysms were defined as ≥ 150% diameter increase of the normal ICA diameter, while saccular aneurysms were defined as a distended sac of any size affecting only part of the ICA circumference.

### Imaging

A 64-slice or 128-slice CT scanner (Philips Brilliance; Philips medical systems, Best, the Netherlands) was used to acquire the CTA scans. The carotid arteries were visualized from the aortic arch to the skull base. Median slice thickness was 0.67 mm (range 0.62–0.90 mm), increment 0.33, collimation 64 × 0.625, and pitch 0.609. Radiation exposure parameters were 100–120 kV and 150–300 mA s. The field of view is set per patient. Injection of 65 ml intravascular contrast (ultravist 300, Schering, Berlin, Germany) was followed by a saline bolus of 40 ml, both at a flow rate of 6 ml per second.

### Software packages

Our study focused on the evaluation of semiautomatic measurement software packages. A search was performed to identify software packages which facilitated semiautomatic vessel tortuosity measurements and were commercially available. To this end, the MEDLINE database was searched using the search terms “software,” “length” or “tortuosity,” “vascular,” and synonyms. Availability of free trial licenses was required in order to participate in this comparative study. Four commonly used commercial software packages were selected: 3mensio Vascular (version 8.1, Pie Medical Imaging BV, Maastricht, the Netherlands), Aquarius iNtuition (version 4.4.12.265, iNtuition Cloud, TeraRecon, Foster City, CA, USA), Vitrea (version 7.4, Vital Images Inc., Toshiba Medical, Minnetonka, MN, USA), and Aycan OsiriX PRO (version 3.10.xxx, Aycan Medical Systems, Rochester, NY, USA). All software packages are commonly used for semiautomatic (vessel) analysis and centerline composition [16–25].

### Study design

Two observers (EEV and VECP) independently scored the 12 datasets at two time points (round 1 and 2, interval ≥ 1 week) with the four software packages. Observers were blinded to each other’s measurements and to earlier measurements with the same software package. For each software package, both observers received a training session by the company of 1 h, and practiced three measurements in order to familiarize with the package and overcome the early learning curve.

### Outcome measures

**Fig. 1:**
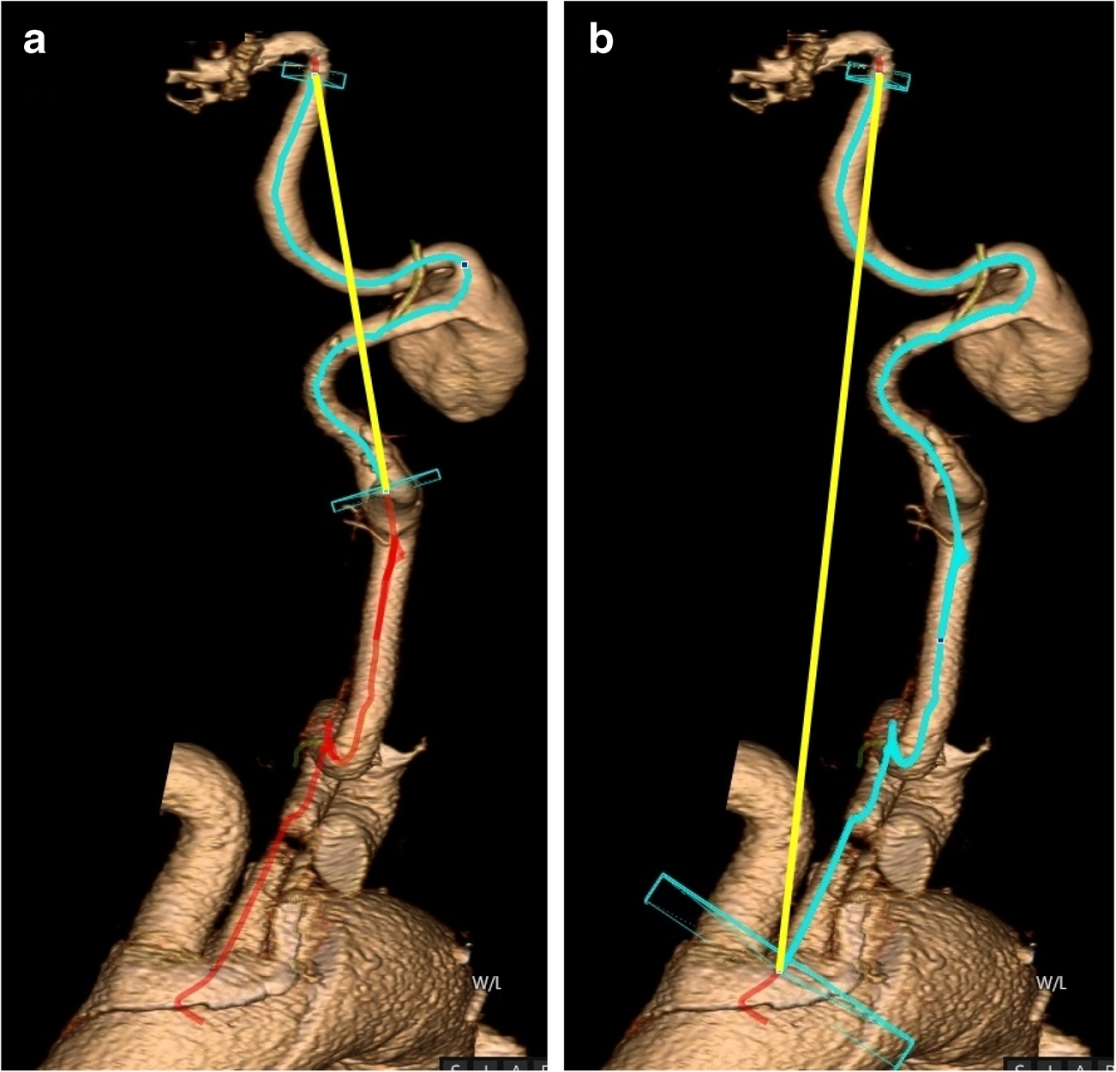
In each dataset, carotid artery tortuosity index (TI) was calculated from the skull base until **a** the carotid bifurcation and **b** the aortic arch (this rendering using Vital Images software). A left internal carotid artery (ICA) with a saccular ECAA is shown, depicted from the aortic arch until the cavernous part of the ICA. The blue line indicates the part of the centerline (in red) that was used to measure the TI of the ICA (**a**) or ICA plus common carotid artery (**b**). This true length was divided by the straight distance (shown in yellow)

In all software programs, carotid artery tortuosity was determined by calculating the tortuosity index (TI) of the carotid artery ipsilateral to the ECAA. The TI was defined as the true length of the central luminal line (CLL) divided by the straight distance. It was calculated in two ways: from the skull base (just proximal from the carotid siphon) until (1) the carotid bifurcation and (2) aortic arch (Fig. 1).

The primary outcome measure was the reproducibility of tortuosity measurements, expressed as the inter- and intra-observer variability in the TI, as measured with the different software programs. The secondary outcome measure was defined as the agreement in absolute TI between the software programs. For both the primary and secondary outcome measures, the correlation between measurements was calculated by using the TI from the skull base to the carotid bifurcation, since ECAAs are located primarily in the ICA. The tertiary outcome measure was the time needed per scan (difference in time between scoring round 1 and 2 (learning curve)).

### Statistics

#### Inter- and intra-observer variability

The intraclass correlation coefficient (ICC) was used to calculate the inter- and intra-observer variability for measurements obtained with one software package (model: two-way mixed, type: consistency). An ICC of 1.0 equals perfect agreement, an ICC of 0.81–0.99 excellent agreement, and an ICC of 0.61–0.80 substantial agreement [26]. The first round of measurements of both investigators was compared in order to calculate inter-observer variability for each software package. Bland-Altman plots were constructed to assess presence of systematic differences between both observers.

#### Agreement on absolute TI

The ICC was also used to calculate agreements on obtained TIs per software package. In order to calculate the differences in measured absolute TI with each software package, the average TI (TI_average_) per case was calculated by taking the average of all four measurements. This was done for each software package separately, thereby producing one TI_average_ per software package for each of the 12 cases.

#### Time needed per scan and learning curve

A Kruskal-Wallis test was used to calculate differences between software packages in time needed to complete all measurements, while a Wilcoxon signed rank test was used to calculate differences in time needed to complete round 1 and 2 within one software package. Mann-Whitney *U* tests were used as post-hoc tests, and Bonferroni correction was applied to account for multiple testing.

Statistical analyses were conducted using SPSS 22.0 (IBM Corp. Released 2013. IBM SPSS Statistics for Windows, Version 22.0. Armonk, NY: IBM Corp.). A *P* value less than 0.05 was considered statistically significant.

## Results

### Patient and aneurysm characteristics

Most ECAA patients were male (*n* = 7; 58%) with a median age of 62 years (range 25–80 years). Aneurysms were saccular (*n* = 7; 58%) or fusiform (*n* = 5; 42%). The median aneurysm diameter reported in the patient records was 12 mm (range 7–40 mm) for saccular aneurysms, and 11 mm (range 5–32 mm) for fusiform aneurysms (Table 1).

**Table 1:** Patient and aneurysm characteristics

Characteristic	ECAA (*n* = 12)
Male	7 (58)
Age (years)	62 (25–80)
Left carotid artery affected	6 (50)
Aneurysm shape	
Saccular	7 (58)
Fusiform	5 (42)
Reported aneurysm diameter (mm)	
Saccular	12 (7–40)
Fusiform	11 (5–32)

Data are given as numbers (percentage) or median (range)

*ECAA* extracranial carotid artery aneurysm

### Reproducibility of measurements

**Fig. 2:**
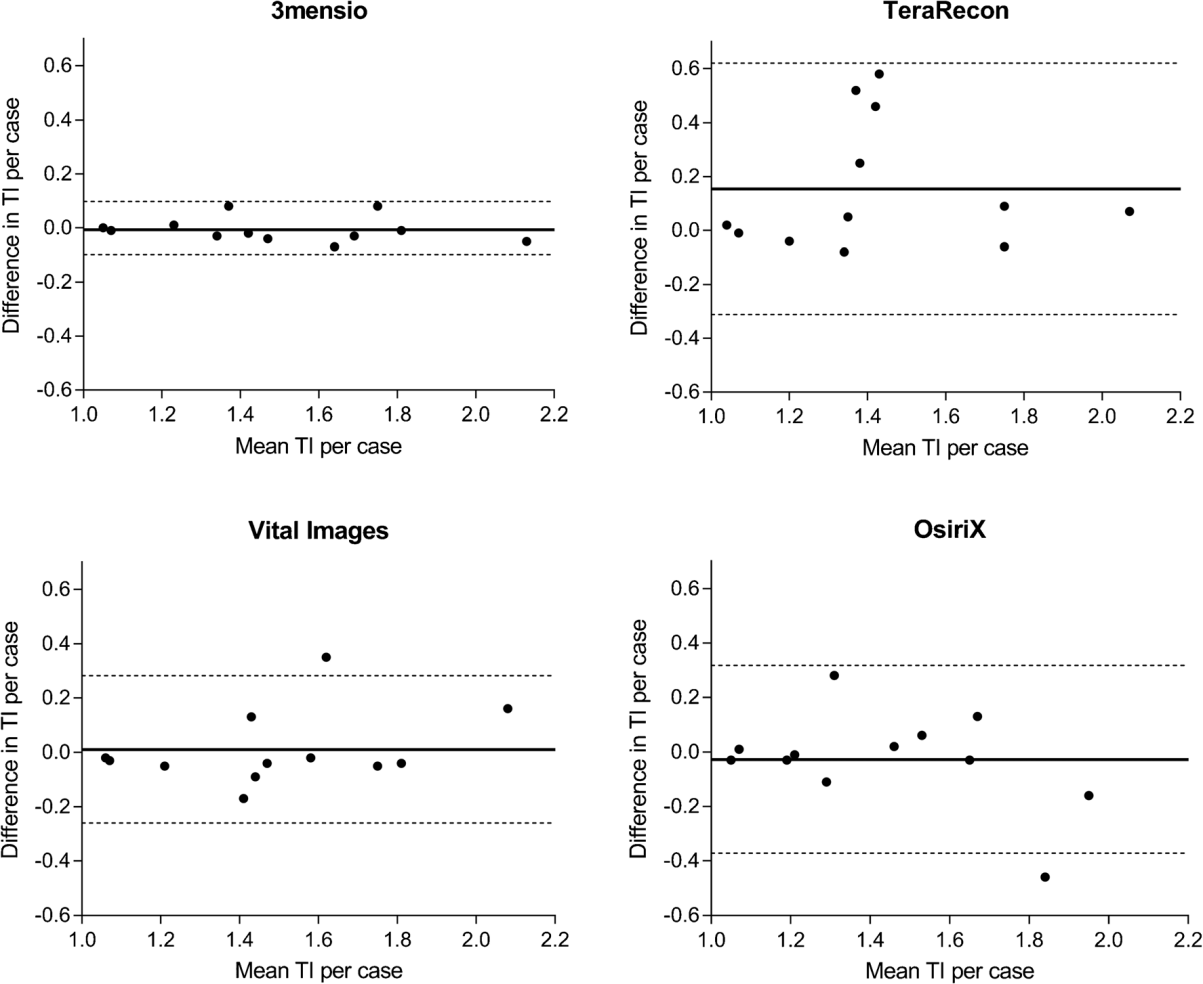
Bland-Altman plots showing agreement of two observers on measured TI for each of the 12 cases. Each graph represents a different software package. The line in the middle represents the mean difference of the TI between the two observers, and the two dotted lines represent the upper and lower limits of agreement (mean difference ± 1.96 × standard deviation)

Inter- and intraclass correlations between software packages are summarized in Table 2. The agreement between both observers was nearly perfect for measurements performed with 3mensio, excellent for Vital Images and OsiriX, and substantial for TeraRecon. Bland and Altman plots were visually evaluated and revealed no clear systematic differences between both observers (Fig. 2). The average intra-observer variability ranged from a perfect agreement of 1.0 for 3mensio to 0.91 for TeraRecon.

**Table 2:** Inter- and intra-observer variability of tortuosity index (TI), measured from the carotid bifurcation to the skull base

	Inter-observer variability	Intra-observer variability (observer 1)	Intra-observer variability (observer 2)	Average intra-observer variability
3mensio	0.99 (0.96–1.0)	1.0 (0.99–1.0)	0.99 (0.96–1.0)	1.0
OsiriX	0.84 (0.53–0.95)	0.95 (0.85–0.99)	0.90 (0.68–0.97)	0.93
TeraRecon	0.72 (0.28–0.91)	0.97 (0.90–0.99)	0.85 (0.55–0.95)*	0.91
Vital Images	0.90 (0.69–0.97)	0.99 (0.97–1.0)	0.97 (0.86–0.99)	0.98

Inter-observer variability was calculated using the first round of measurement of both observers. The average intra-observer variability was calculated as the average ICC of observer 1 and 2

Values are intraclass correlation coefficient (ICC) with 95% confidence interval (CI)

^*^ICC based on 9 out of 12 cases

### Comparison of measured tortuosity indices

In order to calculate the differences in absolute measured TI_average_ with each software package, the ICC was calculated for each software comparison (Table 3). Agreement on measured average TIs between all four packages was excellent, as the ICC for these comparisons equaled 0.95 or higher. Thus, all software packages measured similar TIs for the 12 cases. The median TI_average_ of the 12 cases was 1.42 (interquartile range [IQR] 1.29–1.65) from the carotid bifurcation until the skull base, versus 1.29 (IQR 1.15–1.45) from the aortic arch until the skull base.

**Table 3:** Intraclass correlation coefficients (ICCs) with 95% confidence intervals (CIs) of for each software package comparison of the average tortuosity indices (TI_average_)

Software comparison	ICC (95% CI)
3mensio vs TeraRecon	0.95 (0.82–0.98)
3mensio vs Vital Images	0.98 (0.91–1.0)
3mensio vs OsiriX	0.99 (0.98–1.0)
TeraRecon vs Vital Images	0.99 (0.96–1.0)
TeraRecon vs OsiriX	0.95 (0.83–0.99)
Vital Images vs OsiriX	0.99 (0.96–1.0)

Of note, in OsiriX software, the straight distance had to be drawn in one and the same sagittal/coronal slice even if the skull base and proximal endpoint (bifurcation or aortic arch) were located in a different slice, which could have led to an overestimation of the TI.

### Usability of the software packages

**Fig. 3:**
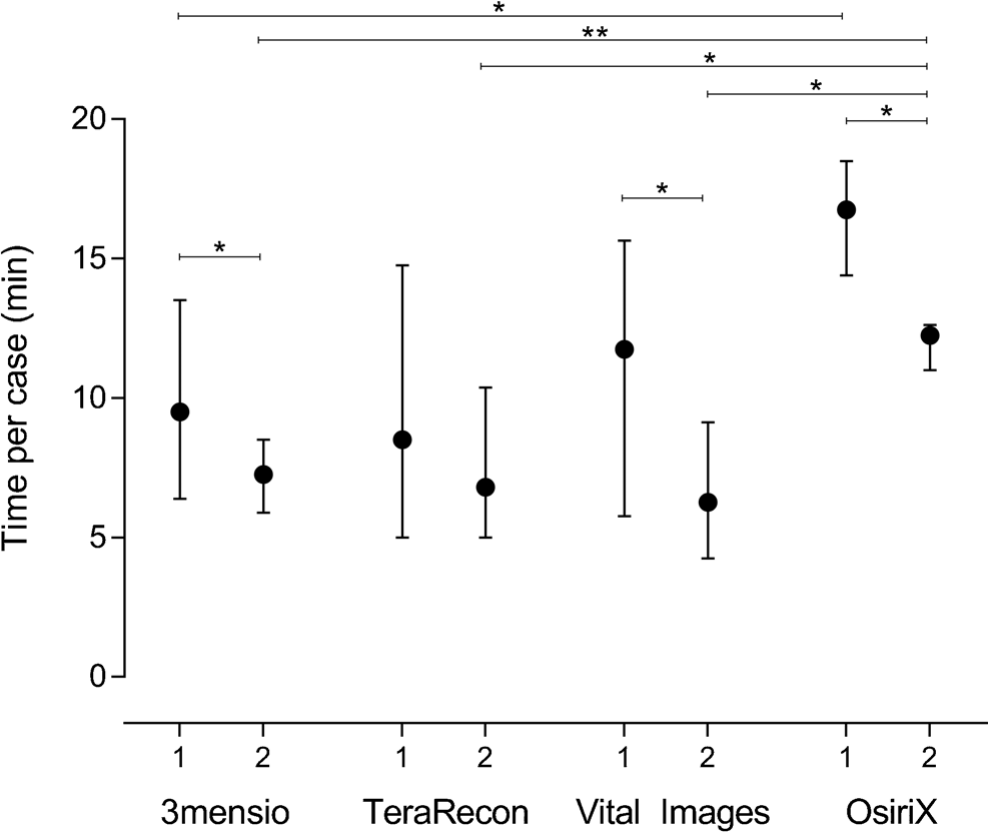
Time needed to complete the first and second round of measurements, for each software package separately. Significant differences are flagged with: *p < 0.008, **p < 0.001. Due to Bonferroni correction for multiple testing, the significance level was set at *p* < 0.008 for comparisons between software packages (Mann-Whitney *U* test), and at *p* < 0.013 for comparisons between round 1 and 2 within a software package (Wilcoxon signed rank test)

The time needed to complete the first round of measurements was comparable between 3mensio, TeraRecon, and Vital Images, with time ranging from a median of 8.5 (IQR 5.0–14.8) minutes for TeraRecon to 11.8 (IQR 5.8–15.6) minutes for Vital Images, while OsiriX software took median 16.8 (IQR 14.4–18.5) minutes (Fig. 3). This difference was significant for both round 1 (*p* = 0.013) and round 2 (*p* < 0.001), and post-hoc tests revealed that OsiriX took significantly longer than the other software packages. Since the number of corrections needed to create a proper fit for the CLL was graded equal for all packages, this difference was probably attributable to a longer time-to-segmentation of the carotid artery for OsiriX software. Except for TeraRecon, round 2 of measurements took significantly shorter compared to round 1. Thus, the usage of the software packages seemed to encompass a significant learning curve (but without influencing reproducibility).

## Discussion

The present study shows that carotid artery tortuosity measurements are reproducible and comparable between the four commercially available software packages that were included in this analysis: 3mensio, TeraRecon, Vital Images, and OsiriX. Calculated inter-observer agreements ranged from 0.99 to 0.72, and agreements between the packages on measured absolute tortuosity indices equaled 0.95 or higher. This suggests that all four software packages can be applied for TI measurements of carotid arteries, and that obtained results with these different software packages are comparable.

A range of different software packages is being used for assessment of vessel anatomies. As clinicians often have limited access to direct comparisons to aid in software selection, it is unclear whether outcomes of these software packages are comparable. To our knowledge, few research groups examined comparability of software packages for measurements on patient vessels. Three papers focused on (phantom) abdominal aneurysm diameter measurements, and demonstrated high levels of agreement for 3mensio, TeraRecon, and Simbionix PRORS software (ICC ≥ 0.82) [16, 17], or TeraRecon versus OsiriX software (ICC ≥ 0.82) [24]. A comparison of 3mensio, TeraRecon, Philips, and Siemens software demonstrated good correlations for semiautomatic carotid stenosis measurements with manual measurements (ICC ≥ 0.81) [19].

However, high vessel tortuosity is likely to challenge semiautomatic centerline composition. We found a median tortuosity index of 1.42 for the internal carotid artery of these ECAA patients, which is deemed higher than the 1.19 of normal internal carotid arteries [18]. Obviously, these data need to be evaluated in more detail in a direct comparison with non-ECAA controls. Nevertheless, the present study shows that even in these challenging vessels, tortuosity measurements remain comparable between software packages. Although fully automated measurements would most likely increase observer agreement and lower time consumption, we believe manual correction of the centerlines will often be required to ensure accuracy of measurements, especially in these tortuous cases.

Currently, little is known about the natural clinical course of patients with an ECAA [5, 27], and no guidelines exist regarding treatment or follow-up. Increased tortuosity values have been linked to increased clinical risk of dissection in different arterial territories [11, 28]. However, whether these vessels were tortuous at baseline and therefore a cause of ECAA formation or merely a consequence remains to be elucidated. Nonetheless, carotid artery tortuosity in ECAA patients has potential as a risk predictor of adverse outcome, and may provide valuable additional sensitivity for the individual ECAA patient’s risk prediction.

There are several limitations to the present study. Due to rarity of disease, only 12 cases could be included in this pilot study. Moreover, merely two observers scored the scans, both with over 1 year of experience with these specific carotid CTA scans. As the primary purpose of this study was to compare the performance of the software programs rather than assessing the true TI values, we consider a good inter-observer reliability between these similarly trained observers most relevant and sufficient. Also, both observers scored the software packages in a different order, but the 12 cases were measured in a non-randomized fashion; therefore, a learning curve might have influenced results obtained for the first and last cases. However, as all packages scored high inter- and intra-observer agreements, this effect may be considered negligible. Finally, it is unknown how little changes in head posture during scanning might influence carotid arteries tortuosity indices. If found relevant, future studies should consider scanning the patients with their head in locked position to rule out confounding due to different head postures.

## Conclusions

In summary, semi-automated carotid artery tortuosity measurements are reproducible and comparable between software packages. Although the reproducibility differed per software packages, all packages scored an acceptable inter-observer agreement. This suggests that the type of software package will not influence outcomes of tortuosity measurements in highly tortuous carotid arteries, and that all four software packages are valid for measuring TI.

## Abbreviations

CCA: Common carotid artery
ECAA: Extracranial carotid artery aneurysm
ICA: Internal carotid artery
ICC: Intraclass correlation coefficient
TI: Tortuosity index
